# Cellular level chemical changes in Scots pine heartwood during incipient brown rot decay

**DOI:** 10.1038/s41598-019-41735-8

**Published:** 2019-03-26

**Authors:** Tiina Belt, Michael Altgen, Mikko Mäkelä, Tuomas Hänninen, Lauri Rautkari

**Affiliations:** 1 Aalto University, School of Chemical Engineering, Department of Bioproducts and Biosystems, P.O.Box 16300, 00076 Aalto, Finland; 2 Swedish University of Agricultural Sciences, Department of Forest Biomaterials and Technology, Skogsmarksgränd, 90183 Umeå, Sweden

## Abstract

The heartwoods of many wood species have natural resistance to wood decay due to the accumulation of antifungal heartwood extractives. The natural durability of heartwoods has been extensively investigated, yet very little information is available on the initiation of heartwood decay. This experiment examined the onset of *Rhodonia placenta* brown rot decay in Scots pine heartwood in order to identify the key changes leading to heartwood decay. An imaging approach based on Raman imaging and multivariate image analysis revealed that the degradation of heartwood began in the innermost cell wall layers and then spread into the remaining cell walls and the middle lamella. Pinosylvins were extensively degraded in the cell walls, middle lamella and extractive deposits, while unidentified material most likely consisting of hemicelluloses and/or lipophilic extractives was removed from the inner cell wall layers. Changes similar to inner cell wall degradation were seen in the remaining cell walls in more advanced decay. The results indicate that the key change in incipient heartwood decay is the degradation of antifungal heartwood extractives. The inner cell wall degradation seen in this experiment may serve a nutritive purpose or facilitate the penetration of degradative agents into the cell walls and middle lamella.

## Introduction

Wood decay is a process in which specialised wood decaying fungi degrade and consume the polymeric constituents of wood. The sapwoods of most wood species are quite susceptible to decay, but many species produce heartwood that can have varying levels of natural decay resistance. Many factors can contribute to this natural durability, but the most important one is generally the presence of antifungal extractives in the heartwood^1^. The heartwoods of many wood species have been characterised in terms of their decay resistance, and the composition and properties of their extractives have been extensively investigated^2–5^. However, the initiation and progress of decay in naturally decay resistant heartwood have received relatively little attention.

In northern Europe, Scots pine (*Pinus sylvestris* L.) is the most widely available wood species that produces naturally decay resistant heartwood. Due to its widespread occurrence and commercial significance, the extent and causes of its decay resistance have been extensively investigated^6–11^. The durability of Scots pine heartwood is known to be moderate or slight on average^12,13^ and primarily due to the heartwood extractives. The phenolic pinosylvins are the most important factor in the decay resistance, and while there are conflicting reports on the significance of resin acids, they are likely to be a contributing factor as well^7–9^. However, very little is still known about the chemical changes that lead to the development of decay in the resistant heartwood material. Many wood decaying fungi are capable of degrading pine heartwood extractives^14–16^, but no information is available on the relationships between extractives degradation and the degradation of other wood constituents. The cellular level distribution of the various chemical changes is also unknown.

With information on heartwood decay lacking, this experiment investigated how the chemical composition of Scots pine heartwood changes during the initial stages of brown rot decay. The main objective was to understand the cellular level progress of decay in the heartwood. The composition of extractives and cell wall polymers was monitored over the course of decay caused by *Rhodonia placenta* (Fr.) Niemelä, K. H. Larss. & Schigel. The cellular level distributions of these changes were then analysed by confocal Raman spectroscopy imaging in combination with principal component analysis (PCA) and cluster analysis. Raman imaging has not been previously applied to the study of brown rot, but it was chosen as the analytical tool in this experiment because it allows the simultaneous spatially resolved monitoring of the wood cell wall polymers^17–19^ and the heartwood extractives^20^. The use of PCA and pixel clustering with Raman imaging improved the sensitivity of the method and made it possible to detect the slight spectral alterations caused by incipient decay.

## Results and Discussion

### Bulk chemical composition

**Figure 1:**
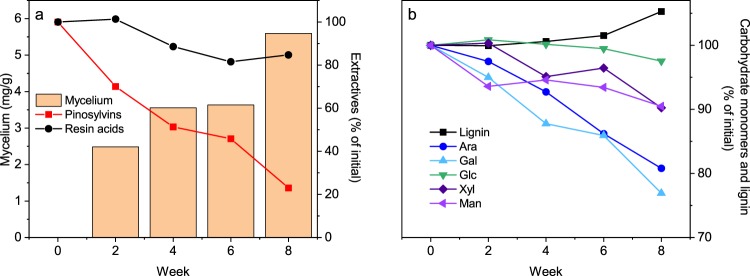
Chemical composition of heartwood degraded by *R. placenta*. Changes in the amount of fungal mycelium and extractives (pinosylvins and resin acids) (**a**) and the relative proportions of carbohydrate monomers and Klason lignin (**b**) over eight weeks of degradation. Replicate samples were pooled for chemical analyses. The values given are an average of two technical replicates. Note the cut y-axis on panel b.

The changes in the chemical composition of decaying heartwood were first analysed by wet chemical methods (Fig. 1). The heartwood samples were extracted and the composition of the extracts analysed by GC-FID, after which the extracted wood samples were acid hydrolysed to determine their lignin content and carbohydrate composition. The fungal mycelium content of the samples was estimated by measuring fungal ergosterol. The most striking change in the composition of heartwood was the degradation of pinosylvins. *R. placenta* was able to cause extensive degradation of the phenolic heartwood extractives, and by week 8, the heartwood contained only 23% of the initial pinosylvins. The two primary heartwood pinosylvins, pinosylvin and pinosylvin monomethyl ether, were degraded at similar rates (see Table S1 in Supplementary Material). The amount of resin acids also decreased over the course of decay, but their degradation was less extensive than that of the pinosylvins. The differences in the extent of pinosylvin and resin acid degradation are most likely due to differences in their toxicity and chemistry. Pinosylvins are more toxic than resin acids^21^ and thus likely targets for the elaborate detoxification system of *R. placenta* that involves laccases, cytochrome P450 monooxygenases and glutathione transferases^22,23^. Pinosylvins are also significantly stronger antioxidants than resin acids^21,24^, which means that they will preferentially react with the wood degrading radicals produced by *R. placenta*^23,25^. Even though the degradation of pinosylvins by various wood decaying fungi has been previously documented^14,15,26^, very little is known about the chemistry of their degradation or the toxicity of their degradation products. However, the rates of pinosylvins degradation and mycelium growth were correlated, suggesting that fungal growth is linked to the elimination of pinosylvins.

In addition to the removal of extractives, *R. placenta* also caused degradation of the wood carbohydrates (Fig. 1b). Arabinose and galactose were the most extensively degraded sugars relative to their initial concentration, followed by xylose and mannose. Glucose was not appreciably degraded, while the relative proportion of Klason lignin increased due to selective carbohydrate removal. The degradation therefore followed a typical incipient brown rot pattern characterised by preferential hemicellulose removal^27,28^. The overall change in the cell wall polymer composition was small (see Table S2 in Supplementary Material), which shows that the wood material was still in early stages of degradation. However, given that brown rot is known to cause extensive disruption of the cell wall structure before substantial changes in composition^27,29^, it is likely that the properties of the cell wall material were already significantly altered.

### Raman imaging and spectral analysis

**Figure 2:**
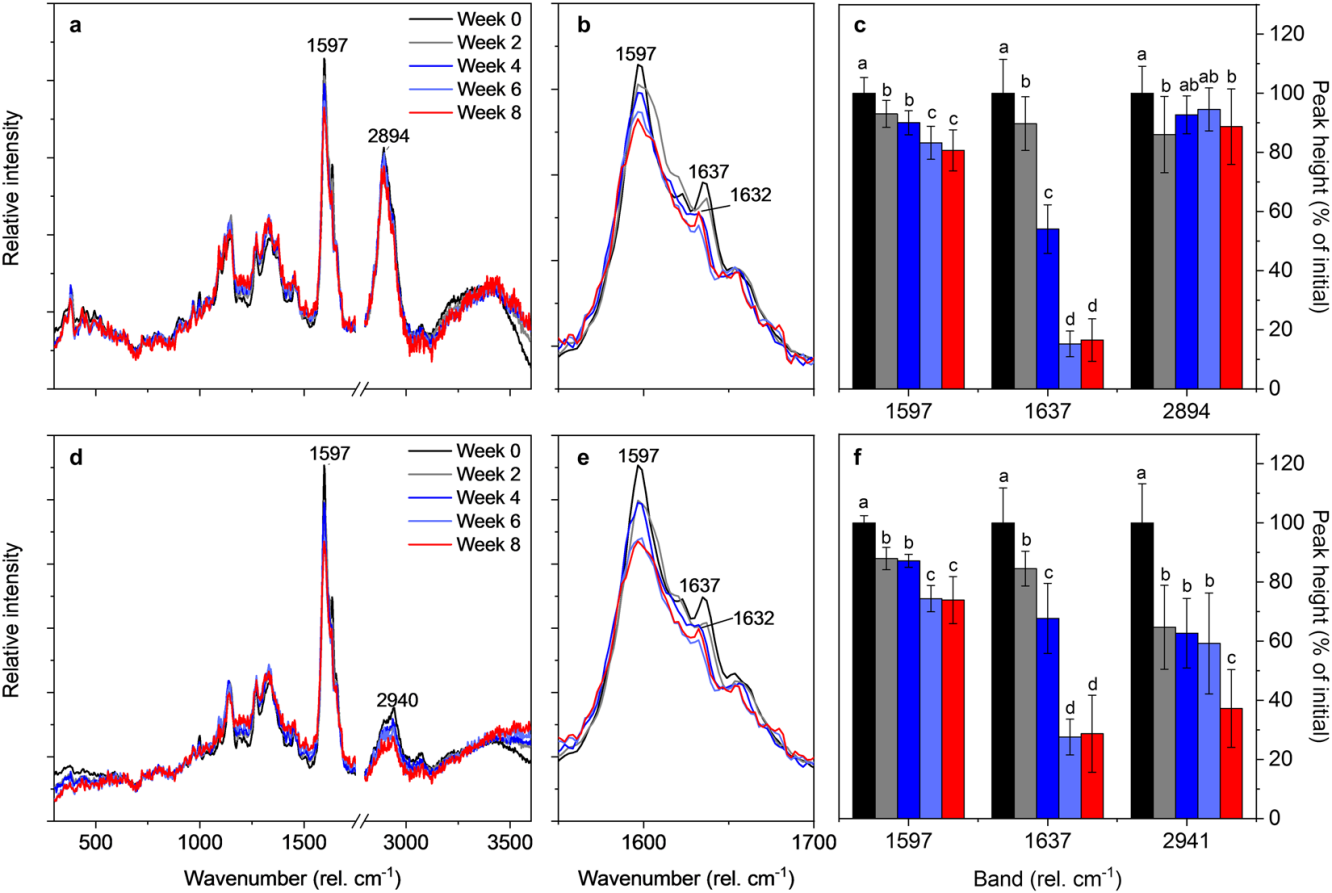
Average Raman spectra of heartwood degraded by *R. placenta*. Average Raman spectra extracted from the S2 cell wall (**a**,**b**) and cell corner (**d**,**e**) regions of Raman images, and the heights of selected bands in the S2 cell wall (**c**) and cell corner (**f**) regions. The cell wall spectra corresponding to each time point were extracted from the cell walls of 16 different cells (four cells on four images), while the cell corner spectra were extracted from the cell corners around 12 different cells (three cells on four images). All spectra have been baseline corrected and normalised by total intensity normalisation. Values with different letters in panels c and f are significantly different from one another (Tukey’s HSD test, p < 0.05).

To investigate how the chemical changes take place on a cellular level, the heartwood samples were subjected to confocal Raman spectroscopy imaging. Raman imaging is a process in which a large number of Raman spectra are collected from the sample at pre-defined positions to build a hyperspectral image in which every pixel contains an entire Raman spectrum. The simplest way to convert the spectral data to images is to integrate the intensity of specific Raman bands. Fig. S3 in Supplementary Material gives an example of a typical integrated intensity Raman image and shows the cellular structures usually seen in Scots pine heartwood samples. In this experiment, integrated intensity Raman images were produced from the degrading heartwood samples, and average spectra were then extracted (see Fig. S3) from the secondary cell wall (S2 layer) and cell corner regions for a closer examination of the spectral changes. The average cell wall and cell corner Raman spectra corresponding to the different stages of decay are given in Fig. 2. The spectra in Fig. 2 have been baseline corrected by deducting the values of a second-order polynomial fitted on the entire wavelength range and normalised by means of a total intensity vector normalisation procedure. Baseline correction and normalisation were necessary for data analysis because brown rot decay caused a substantial increase in the fluorescent background of the spectra (see Fig. S4 in Supplementary Material). The noisiness of the spectra increased as well, particularly between week 6 and week 8. Together with the increasing fluorescence, the decreasing signal-to-noise ratio limits the applicability of the current method to the incipient stages of brown rot decay.

The S2 cell wall and cell corner Raman spectra of Scots pine heartwood were not drastically altered by incipient brown rot decay, but some changes were nonetheless visible, particularly in the 1550–1700 cm^−1^ region (Fig. 2b,e). One of the most significant changes was the reduction in band intensity at 1637 cm^−1^. The 1637 cm^−1^ band corresponds to the C=C stretch of pinosylvins^20,30^, and its intensity decreased to 17% of initial in the cell wall regions and 29% of initial in the cell corner regions by week 8 (Fig. 2c,f). From week 4 onwards a new band could be seen next to the pinosylvins band at 1632 cm^−1^, most likely representing the accumulation of some heartwood degradation product. The intensity of the aromatic ring stretch band at 1600 cm^−1^ also decreased over the course of decay. The decrease was slightly stronger in the cell corner than cell wall regions, with cell corner intensity reduced to 74% of initial and cell wall intensity to 81% of initial by week 8. The aromatic ring stretch band is usually considered a lignin-derived band, but in Scots pine heartwood it also includes contributions from pinosylvins^20,30–32^. The decrease in its intensity is likely to be due to the degradation of pinosylvins, given that the relative amount of lignin increased due to *R. placenta* decay (Fig. 1b). Decay-induced changes in the chemistry of lignin are unlikely to contribute to the intensity decrease because the modifications typically associated with brown rot have no effect on the aromatic content of lignin^33,34^. Previous research has in fact demonstrated that the intensity of the aromatic ring stretch band increases due to brown rot in wood that is free from phenolic heartwood extractives^35^.

In addition to the bands at 1550–1700 cm^−1^, changes could also be seen in the CH and CH_2_ stretching region at 2800–3000 cm^−1^. The CH/CH_2_ stretching region consists of many overlapping bands, including strong carbohydrate bands at 2820–2970 cm^−1^, strong resin acid and fatty acid bands at 2800–3070 cm^−1^, and medium lignin bands at 2845–3070 cm^−1^ ^31,32,36–38^. The intensity of the CH/CH_2_ stretching bands decreased slightly in the S2 cell wall layer over the course of decay (Fig. 2a,c), but the trend was inconsistent and the variations within and between samples were large. The intensity decrease was stronger in the cell corner region (Fig. 2b,f), where a particularly strong decrease was seen at the spectral region corresponding to the asymmetric CH stretch of lignin methoxy groups (2940 cm^−1^)^38^. The decrease in band intensity at 2940 cm^−1^ is consistent with lignin demethylation, which is a typical feature of brown rot decay^33,34^. However, given the unspecific nature of the CH/CH_2_ stretching bands, the changes in the 2800–3000 cm^−1^ region may also include contributions from other lignin modifications or the degradation of carbohydrates or resin acids. Finally, slight changes were also detected in the intensity of several lignin and cellulose derived finger print bands (1091, 1149, 1271, 1330, 1377 cm^−1^)^31,36–38^, but given the small magnitude of the changes, their intensities were not analysed in more detail.

**Figure 3:**
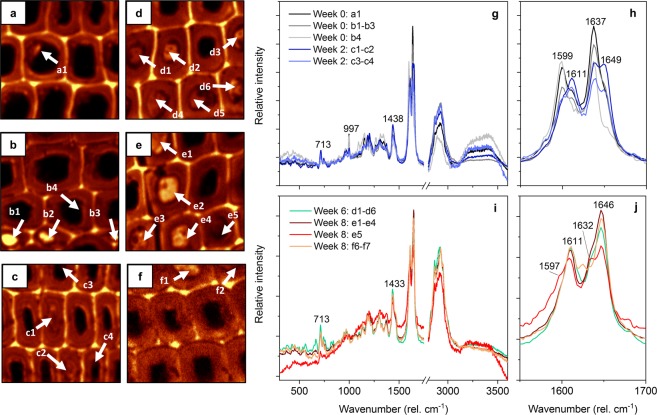
Extractive deposits in heartwood degraded by *R. placenta*. Raman images of samples degraded for 0 (**a**,**b**), 2 (**c**), 6 (**d**) and 8 (**e**,**f**) weeks produced by integrating lignin and extractive bands at 1550–1700 cm^−1^, and the average Raman spectra of the week 0 and 2 (**g**,**h**) and the week 6 and 8 deposits (**i**,**j**). Deposits (arrows) are identified by their letter and number. All spectra have been baseline corrected and normalised by total intensity normalisation.

Interesting changes could also be seen in the Raman spectra of extractive deposits. Extractive deposits are a typical feature of Scots pine heartwood and include small deposits consisting almost entirely of pinosylvins and filled tracheid lumens containing a mixture of pinosylvins and resin acids^20^. Figure 3 shows some of the extractive deposits found in the Scots pine heartwood samples (Fig. 3a–f), along with their average Raman spectra (Fig. 3g–j). The small deposits and the filled lumens seen in the week 0 samples (Fig. 3a,b) appeared to be rich in pinosylvins. Their spectra were dominated by the aromatic ring stretch band at 1600 cm^−1^ and the pinosylvin C=C stretch band at 1637 cm^−1^. An additional band could be seen at 997 cm^−1^, representing the 1,3,5-substituted aromatic ring of pinosylvins^20,30,32^. The intensity of the pinosylvins bands decreased in the week 2 deposits (Fig. 3c), while the intensity of bands derived from resin acids and other lipophilic extractives increased. Particularly strong bands corresponding to the aromatic ring stretch of dehydroabietic acid and the C=C stretch of abietane-type resin acids and fatty acids were seen at 1612 and 1650 cm^−1^, respectively^20,32,39^. The intensity of resin acid and fatty acid derived bands at 1438 cm^−1^ and 713 cm^−1 ^^32,39^ increased as well. The change in the composition of deposits from week 0 to week 2 is likely to be due to the degradation of pinosylvins, although local variations in the composition of deposits may also account for some of the differences. The removal of pinosylvins from the extractive deposits became extensive in the more advanced stages of decay. No small deposits were detected in the week 6 and week 8 samples, and the filled lumens seen in the samples (Fig. 3d–f) were almost entirely devoid of pinosylvins, as evidenced by the lack of pinosylvins-derived bands at 1600 and 1637 cm^−1^. Strong resin and fatty acid bands were still seen at 1612 and 1650 cm^−1^ as well as 1433 and 713 cm^−1^. The changes in the composition of extractive deposits are therefore in agreement with the overall change in extractive composition (Fig. 1a).

### PCA and cellular level distributions

The data presented in Figs. 1–3 provide information on the chemical changes taking place in heartwood during brown rot decay. To gain more insight into the fine cellular level distribution of these changes, the Raman imaging approach was combined with PCA. PCA is a mathematical procedure that summarises information from hundreds of wavelengths in just a few uncorrelated variables called principal components (PCs). The PCs are obtained through an axis rotation that maximises variation explained in the data. The loadings illustrate spectral changes based on the PCs, and the scores describe differences between the individual pixels. With images the initial score vectors can also be refolded back to image dimensions to allow the visualisation of spatial variation in the samples. In general, PCA is more sensitive than the traditional integrated intensity-based imaging approach because it can use the entire spectrum to look for differences between image pixels rather than just one or a few different bands. PCA also reduces the noisiness of data by excluding variation caused by measurement uncertainties.

To utilise PCA in analysing the chemical changes caused by brown rot decay, five heartwood Raman images (one per each stage of decay) were selected and combined into an image mosaic. Images with extractive deposits were selected where possible given the frequent occurrence of deposits in Scots pine heartwood and their potential significance in decay resistance. Finally, as the largest variation in the Raman images was related to differences between the cell walls and the lumen, pixels representing lumen water were excluded from the final PCA. This simplified the interpretation of the PCs and made it easier to detect detailed changes in the cell walls.

**Figure 4:**
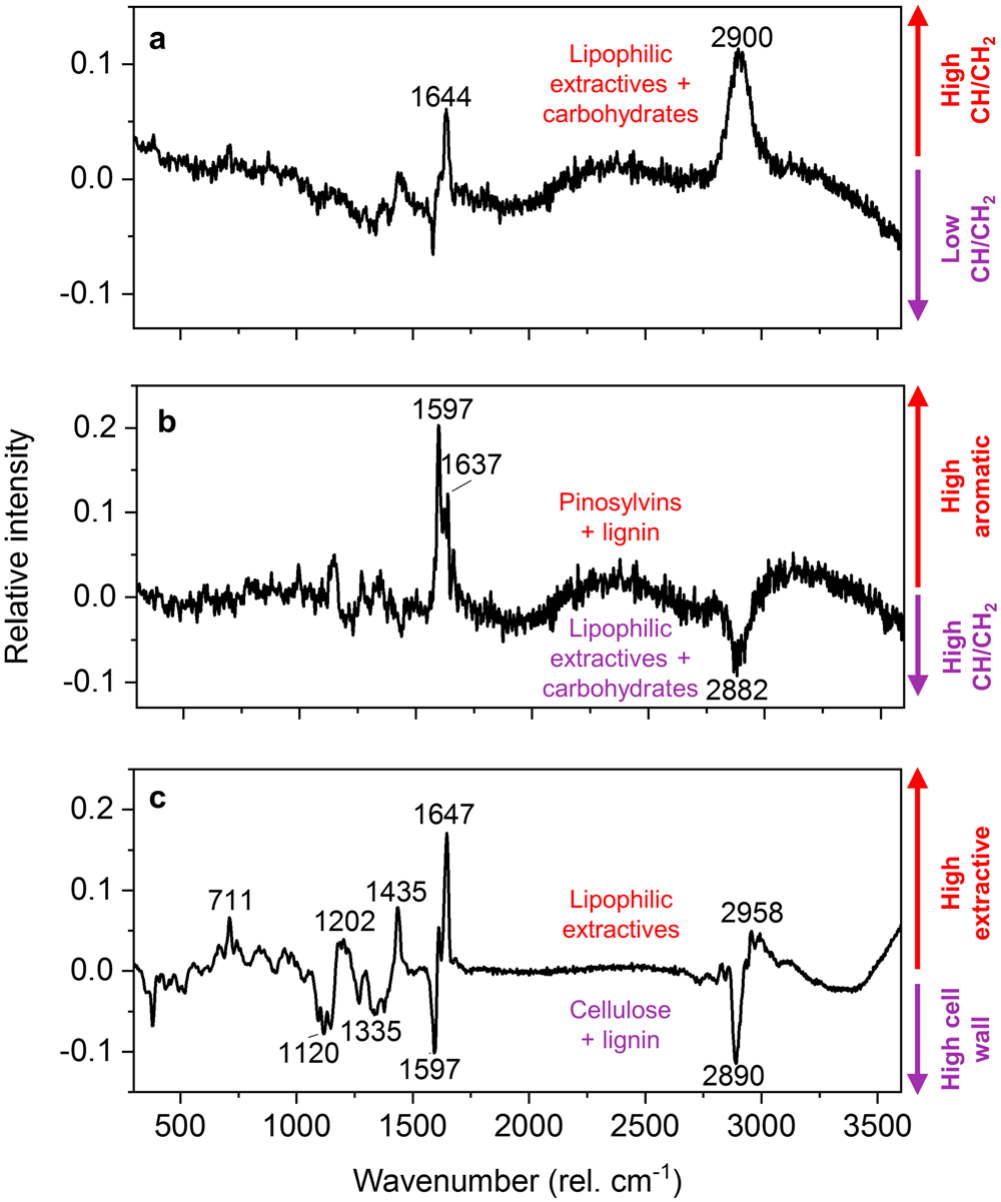
PCA loading plots. PC 1 (**a**) separates data according to CH/CH_2_ stretching intensity (derived from carbohydrates and lipophilic extractives), while PC 2 (**b**) separates the data according to aromatic contributions (pinosylvins and lignin) and CH/CH_2_ stretching intensity (carbohydrates and lipophilic extractives). PC3 (**c**) separates the data according to contributions from lipophilic extractives and cell wall polymers (cellulose and lignin).

The loadings of the first three PCs are presented in Fig. 4. PC 1 accounted for 38% of the total variation within and between the Raman images and was dominated by a strong positive band at 2900 cm^−1^ that spanned the entire CH/CH_2_ stretching region. Smaller positive bands derived from resin acids and fatty acids (lipophilic extractives) were present at 1644 cm^−1^ and 1435 cm^−1^, while a small negative band was found at the aromatic ring stretch region at 1585 cm^−1^. PC 1 can therefore be considered a representation of mainly the lipophilic extractives and other CH/CH_2_ stretch-producing material. PC 2 accounted for 22% of the total variation and featured strong positive bands at 1597 cm^−1^ (aromatic ring stretch) and 1637 cm^−1^ (C=C stretch of pinosylvins). PC 2 also had a negative band at 2882 cm^−1^ that spanned the entire CH/CH_2_ stretching region. Given that the contributions of pinosylvins and lignin to the aromatic ring stretch band cannot be separated, PC 2 can be considered a representation of pinosylvins and aromatic lignin structures (positive) and CH/CH_2_ stretch-producing material such as carbohydrates and lipophilic extractives (negative). The third PC (12% of the total variation) showed positive bands at 1647, 1435, 1202, 711 and 2958 cm^−1^, all of which can be attributed to resin acids and other lipophilic extractives^20,32,39^. Negative bands corresponding to lignin or wood carbohydrates were found at 2890, 1597 and in the fingerprint region^31,36–38^. PC 3 therefore separates extractives from the cell wall polymers. PCs 4 and onwards explained only a few percent or less of the total variation and provided little meaningful chemical information.

**Figure 5:**
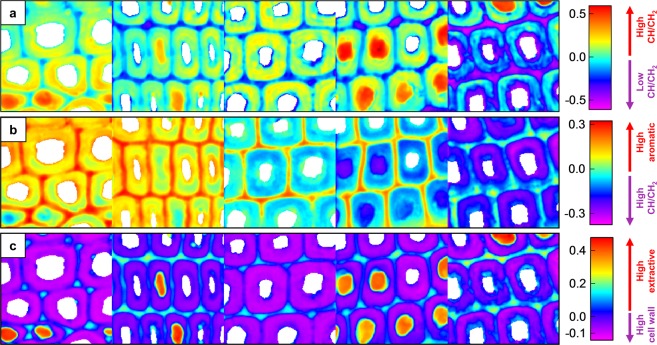
PCA score images. The PC 1 scores (**a**) separate the data into pixels with high or low CH/CH_2_ stretching intensity, while the PC 2 scores (**b**) separate the data into pixels with high aromatic contribution or high CH/CH_2_ stretching intensity. The PC 3 scores (**c**) separate the data into pixels with high contributions from lipophilic extractives or cell wall polymers. Samples from left to right are week 0, 2, 4, 6 and 8. The size of each individual sample image is 70 × 70 μm.

The score images corresponding to PCs 1–3 are shown in Fig. 5. In the case of PC 1 (Fig. 5a), the pixels with positive score values are those in which the Raman spectrum has strong contributions from CH/CH_2_ stretch-producing material such as lipophilic extractives and carbohydrates, while the opposite is true for the pixels with negative scores. PC 1 indicated higher positive scores in the cell wall regions than in the middle lamella/cell corners in all samples, reflecting the higher concentration of carbohydrates in the cell walls. The cell wall scores showed variation within each sample, possibly caused by the penetration of lipophilic extractives into the cell wall. However, given the unspecific nature of the CH/CH_2_ stretching bands and the occurrence of high PC 1 scores in the cell walls of some deposit-free cells, the variations cannot be reliably ascribed to just lipophilic extractives. Over the first six weeks of degradation, the heartwood cell walls showed little degradation of material corresponding to the positive PC 1 loading bands, apart from the appearance of a narrow zone of degradation in the S3 layer. Cell wall degradation became noticeable in the week 8 sample, where particularly intense degradation was seen in the S3 layer of the cell wall. The middle lamella/cell corner regions were also degraded. Although brown rot decay is usually thought to involve mainly the S2 layer of the cell wall^40^, degradation of the S3 layer and the middle lamella have been previously documented in incipient *R. placenta* decay^41–43^. The incipient decay of these regions is characterised by the degradation of easily digestible polysaccharides such as pectins and hemicelluloses^42^. However, the degradation revealed by the PC 1 scores may also involve lignin modification or the degradation of lipophilic extractives. The degradation of the inner cell wall layers may facilitate the penetration of fungal degradative agents into the cell walls, particularly if the removed material is lipophilic in nature.

In the case of PC 2 (Fig. 5b), the pixels with positive score values represented areas of high pinosylvins and lignin concentration, while the pixels with negative scores indicated areas with a high concentration of CH/CH_2_ stretch-producing material such as carbohydrates and lipophilic extractives. Over the course of decay, material corresponding to the positive loading plot bands was removed from the heartwood samples. The removed material was likely to be pinosylvins, given that the relative amount of lignin actually increased over the course of decay (Fig. 1b). Pinosylvins were removed from the cell wall and middle lamella regions at fairly similar rates. Some local variations in score values could be seen within the cell walls, but a comparison with the PC 1 score images revealed that the spots of lower PC 2 scores coincided with spots of more positive PC 1 scores. This suggests that the local score variations seen in Fig. 5b were not caused by an uneven distribution of residual pinosylvins but an uneven distribution of CH/CH_2_ stretch-producing material.

In the case of PC 3 (Fig. 5c), the positive score values indicated areas of high resin acid concentration, while the negative scores indicated areas with strong contributions from carbohydrates and lignin. The PC 3 scores effectively separated the lumen-filling extractive deposits from the cell walls and the middle lamellae. Contrary to PC 1, the PC 3 scores suggested no penetration of resin acids or other lipophilic extractives into the cell walls. The PC 3 scores also showed very little variation over the course of decay, most likely reflecting the relatively low extent of resin acid degradation (Fig. 1a) and their persistence in the extractive deposits (Fig. 3).

**Figure 6:**
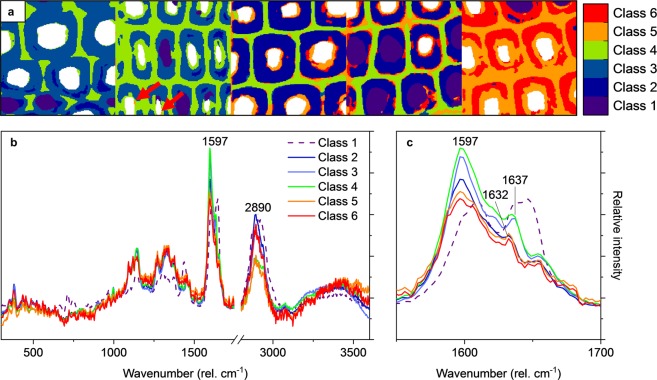
PCA-based cluster analysis. A cluster image based on six classes (**a**) and the average spectra of the different classes before mean-centering (**b**,**c**). Class 1 represents extractive deposits while classes 2–6 represent the cell walls and middle lamellae in different stages of degradation. The red arrows in (**a**) point to spots of more advanced degradation in the week 2 sample.

The PC 1–3 scores provided an effective grouping for the thousands of Raman image pixels (see Fig. S5 in Supplementary Material). To make use of the effective grouping, the pixels were further clustered using partitional K-means based on the PC scores. The number of clusters was chosen based on the mean correlation of pixels with the respective cluster centroids for a specific number of clusters. As illustrated in Fig. S6 in Supplementary Material, little decrease in mean correlation was observed after 6 clusters. The number of cluster was therefore set to 6. Higher class assignments were also tested but found to provide little additional information on heartwood degradation. As shown in Fig. 6, the degradation of heartwood started in the innermost cell wall layers. The first signs of degradation were seen in the week 2 sample, where an area characterised by reduced CH/CH_2_ stretch intensity (class 4) appeared in the S3 layer of the cell wall. Additional spots of more advanced degradation (reduced band intensity at 2890, 1637 and 1600 cm^−1^, class 5) could be seen on the lumen walls of some cells. By week 4, the spots of more advanced degradation (class 5) had grown to encompass the whole S3 layer of each cell, and areas of even further degradation (class 6) could be seen in the S3/S2 layer of some cells. The S2 layer of the week 4 cell walls also had reduced band intensity at 1600 cm^−1^ and 1637 cm^−1^ (class 2), suggesting that the removal of CH/CH_2_ stretch-producing material from the S3 layer is followed by the degradation of pinosylvins in the whole cell wall. Isolated spots of degradation (class 5 and class 6) also appeared in the middle lamella/outer cell wall layers.

The degradation pattern seen in the week 6 sample was similar to that of week 4. However, the degradation of pinosylvins (class 5) had progressed further into the middle lamella, and signs of advanced cell wall degradation (class 6) could also be seen in the outer layers of many cell walls. The inner cell wall layers of the deposit-containing cells showed less degradation than the inner layers of the deposit-free cells, but it is unclear whether this difference is due to a higher initial concentration of extractives or reduced degradation due to the presence of the deposit. Regardless of the cause of the difference, the middle lamellae and the outer cell wall layers of the deposit-containing cells still showed signs of degradation.

As expected, the week 8 sample showed the most advanced decay. The average spectrum of the entire middle lamella region had strongly reduced band intensity at 1637 and 1600 cm^−1^ (class 5), consistent with advanced pinosylvins degradation. The cell walls also had strongly reduced intensity at 1637 and 1600 cm^−1^ (class 5 and class 6), apart from a few isolated spots of lesser degradation. As in the earlier stages of decay, the inner cell wall layers showed more extensive changes than the middle layers. The inner layers (S3 and inner S2) of all deposit-free cells had lower CH/CH_2_ stretch intensity than the middle S2 layers (class 5 in the outer layers vs. class 6 in the middle layers), and in some cells the low intensity areas extended through the entire cell wall into the middle lamella. The CH/CH_2_ stretch intensity of the middle cell wall layers was reduced compared to earlier weeks, suggesting that the degradation of pinosylvins in the cell walls is followed by the degradation of CH/CH_2_ stretch-producing material. The degradation of Scots pine heartwood by *R. placenta* therefore appears to start with the degradation of the inner cell wall layers, followed by the degradation of pinosylvins in the cell wall and middle lamella regions and finally the degradation of CH/CH_2_ stretch-producing material in the remaining cell walls. The initiation of decay in the inner cell wall may be connected to the lower porosity^44^ and higher extractive content^20,32^ of the S3 cell wall layer that are likely to limit the penetration of fungal degradative agents into the cell walls. The degradation of pinosylvins in the next step is likely to reduce the toxicity of the wood material and decrease the antioxidant activity of the extractives, allowing the fungus to further colonise the wood material and attack the cell wall polymers with wood degrading radicals.

## Conclusions

This work investigated the incipient stages of brown rot decay in Scots pine heartwood in order to identify the key steps in heartwood degradation. The Raman spectroscopy-based experimental approach showed that the most substantial spectral changes were related to reductions in the intensity of pinosylvins-derived bands and the CH/CH_2_ stretching band. Although not assigned to any one heartwood chemical component, the changes in CH/CH_2_ stretching intensity were most likely due to the degradation of hemicelluloses and/or resin acids and other lipophilic extractives. Bulk chemical analyses confirmed the degradation of hemicelluloses, resin acids and pinosylvins over the course of heartwood decay. The PCA score images and cluster analysis revealed that heartwood degradation began in the innermost cell wall layers and then spread into the remaining cell walls and the middle lamella. CH/CH_2_ stretch-producing material was first degraded in the inner cell wall layers, after which pinosylvins were removed from the cell walls and middle lamella. The attack on the inner cell wall layers may facilitate the penetration of degradative agents into the cell walls. In the final stage of decay examined in this experiment, slight degradation of CH/CH_2_ stretch-producing material was also seen in the whole cell wall region, suggesting that the degradation of pinosylvins is followed by other degradative reactions.

## Methods

### Sample preparation

The heartwood samples were prepared from one fresh log of 70 year old Scots pine wood that was stored frozen and protected from light and desiccation until use. A disc approx. 250 mm in thickness was sawn from the log, after which sticks measuring the full 250 mm in length were sawn from the outer edges of heartwood (see Fig. S7 in Supplementary Material). The sticks were planed to a cross-section of 7 mm × 7 mm and then sawn to produce a series of final sample sticks measuring 40 mm in length. The sample sticks were sealed in plastic bags immediately after cutting and stored frozen until sterilisation by irradiation (25–50 kGy dose).

### Decay test

The heartwood samples were exposed to the brown rot fungus *Rhodonia placenta* (strain BAM 113, Federal institute for materials research and testing culture collection, Germany), which was maintained on 2% malt extract agar. Decay testing was conducted on 2% malt extract agar plates, which were inoculated with plugs of mycelium from the growing edges of the stock cultures and incubated until the fungus had covered the surface of the agar. The sterilised wood sample sticks were then placed on the plates on top of sterile filter paper supports, with five randomly selected sticks per plate. The plates were incubated at room temperature in the dark, with one plate withdrawn for sampling after 0, 2, 4, 6, and 8 weeks of incubation. Surface mycelium was removed from the sample sticks before their use in analyses.

### Raman imaging and image analysis

Every sampling week, Raman imaging samples were prepared from two randomly selected sample sticks. Cross-sections (20–50 µm in thickness) were cut from the ends of the sticks with a rotary microtome, after which the cross-sections were placed on objective slides with a few drops of water, covered with cover slips and edge sealed with nail polish. All cross-sections were analysed within two days of sampling. Raman images were acquired using a WITec alpha 300 RA Raman microscope equipped with a 532 nm frequency doubled Nd:YAG laser (used at 30 mW), a 20x air objective (NA = 0.4), and a DU970-BV EMCCD camera behind a 600 lines mm^−1^ grating. The size of each image was 70 μm × 70 μm, with 150 lines per image and 150 points per line. An integration time of 0.3 s was used. At least two separate scans were acquired from the latewood regions of each section. False-colour images describing the concentration of specific chemical components were generated in the WITec ProjectPlus software by integration of specific bands in the Raman spectrum. Background was subtracted from the integrated intensity by setting the spectral intensity to zero at four wavelengths above and below the wavenumber range of interest. Average cell wall, cell corner and extractive deposit spectra were obtained by drawing squares on the regions of interest to extract the average spectrum of the selected areas. Average cell wall spectra were extracted from the cell walls of four different cells on each image, while average cell corner spectra were extracted from the cell corners around three different cells.

To analyse spectral changes, the extracted average spectra were baseline corrected to remove fluorescence and normalised to decrease the intensity differences between spectra (see Fig. S4 in Supplementary Material). Raman shifts outside 300–3600 cm^−1^ were excluded as they mainly consisted of noise. Baseline correction was performed by fitting a second-order polynomial over the selected wavenumber range, while vector normalisation was performed by calculating the sum of the squared intensity values of the spectrum and using the squared root of this sum as the normalisation constant^45^. Additional random spike (cosmic ray) removal was performed with a moving window of 3 × 3 pixels by replacing outlier spectra with the median spectrum of each window. Band heights were determined for bands of interest to provide quantitative data on the spectral changes. To obtain the height of the shoulder band at 1637 cm^−1^, the bands in the spectral region 1550–1700 cm^−1^ were deconvoluted using a Gaussian function.

For PCA the individual Raman spectra were baseline corrected and normalised as above. The pre-processed Raman images were combined into an image mosaic, which was unfolded into a two-dimensional array with individual pixels as row objects and wavenumbers as the corresponding columns. The data were then decomposed into a bilinear combination of pixel scores and wavenumber loadings according to principal component (PC) model after mean-centering^46^. Non-hierarchical image clustering was performed using partitional K-means based on PC scores. A predetermined number of cluster centroids were chosen based on the mean correlation of pixels with the respective cluster centroids for a specific number of clusters (see Fig. S6 in Supplementary Material). Clusters smaller than one percent in area were not allowed to be assigned as a unique class. Data analysis and plotting were performed with the Matlab® (The Mathworks, Inc.), PLS Toolbox (Eigenvector Research, Inc.) and OriginPro (OriginLab Corp.) software packages.

### Bulk chemical analyses

After cutting samples for Raman imaging, the remaining sample sticks from each week were combined, splintered, and freeze-dried. The freeze-dried wood material was ground in a Wiley mill (mesh 20) and used for the analysis of bulk chemical composition and the estimation of fungal growth. To determine the composition of extractives, 0.3 g of the wood powder was extracted twice with 5 ml of MeOH in a sonicator (45 °C, 30 min per extraction). Aliquots of both extracts and internal standard solution (heneicosanoic acid) were evaporated to dryness under vacuum, redissolved in 500 µl of dry pyridine, and then silylated at 70 °C for 60 min after the addition of 400 µl of N,O-bis(trimethylsilyl)trifluoroacetamide and 100 µl of chlorotrimethylsilane. The composition of extractives was analysed by GC-FID (Shimadzu GC 2010) using an HP-5 column (30 m × 0.32 mm i.d., 0.25 µm film thickness) as previously described^47^. The oven temperature program was 2 min at 100 °C, 6 °C min^−1^ to 280 °C, and 5 min at 280 °C. To determine the composition of cell wall polymers, 0.3 g of the extracted wood powder was acid hydrolysed according to NREL/TP-510-42618. The amount of Klason lignin in the hydrolysates was determined gravimetrically. The composition of monosaccharides was determined by HPAEC-PAD (Dionex ICS-3000) using a CarboPac PA20 column and water as eluent (0.37 ml min^−1^) as described before^47^. The chemical analyses were performed in duplicate.

To estimate fungal growth, the ergosterol content of the wood material was determined. Ergosterol was extracted following the method of Niemenmaa *et al*.^48^. Briefly, 0.25 g of wood powder was saponified with 3 ml of 10% KOH in MeOH (60 min at 80 °C). After the addition of 1 ml of water, ergosterol was extracted from the solution with n-hexane (2 × 2 ml). A 3 ml portion of the combined hexane phases was evaporated to dryness under vacuum, redissolved in dry pyridine, and silylated for 20 min at 70 °C after the addition of 400 µl of N,O-bis(trimethylsilyl)trifluoroacetamide and 100 µl of chlorotrimethylsilane. The ergosterol content of the samples was quantified by GC-FID against an ergosterol calibration curve using the same column as above. The oven temperature program was set to 2 min at 200 °C, 10 °C min^−1^ to 300 °C, and 15 min at 300 °C. Helium was used as the carrier gas at 1 ml min^−1^. The ergosterol content of the fungal mycelium scraped off the sample surfaces was also determined using the same procedure. Fungal growth was then expressed as mycelium content (mg of mycelium per g of wood). The ergosterol determinations were performed in duplicate.

## Supplementary information

Supplementary material

## Data Availability

The datasets generated during and/or analysed during the current study are available from the corresponding author on reasonable request.
