# Supplementary material for: Cellular level chemical changes in Scots pine heartwood during incipient brown rot decay
Source: Sci Rep. 2019 Mar 26;9:5188. doi: 10.1038/s41598-019-41735-8 (PMC6435796; doi:10.1038/s41598-019-41735-8)
Supplement: Supplementary file 1 — Supplementary material [file 41598_2019_41735_MOESM1_ESM.pdf]

Supplementary material

Cellular level chemical changes in Scots pine heartwood during incipient brown rot decay

Tiina Belt[a]*, Michael Altgen[a], Mikko Mäkelä[a,b], Tuomas Hänninen[a], Lauri Rautkari[a]

[a]Aalto University, School of Chemical Engineering, Department of Bioproducts and Biosystems, P.O.Box 16300, 00076 Aalto, Finland

[b]Swedish University of Agricultural Sciences, Department of Forest Biomaterials and Technology, Skogsmarksgränd, 90183 Umeå, Sweden

*Corresponding author, tiina.belt@aalto.fi

Supplementary Table S1. Extractive composition of Scots pine heartwood over 8 weeks of degradation by *R. placenta* (mg/g oven dry wood). PS, pinosylvin; PSM, pinosylvin monomethyl ether; RA 1-8, resin acids 1-8

	**Week 0**	**Week 2**	**Week 4**	**Week 6**	**Week 8**
**PS**	7.7	5.3	3.7	3.3	1.7
**PSM**	11.2	7.9	6.0	5.4	2.6
**RA 1**	8.8	9.2	8.0	7.6	7.9
**RA 2**	1.3	1.4	0.0	0.0	0.0
**RA 3**	2.2	2.4	2.1	1.9	2.1
**RA 4**	15.2	15.7	13.9	13.0	13.3
**RA 5**	17.1	18.8	15.0	12.7	13.7
**RA 6**	11.4	12.4	11.0	11.0	10.8
**RA 7**	14.2	13.0	12.3	11.3	11.8
**RA 8**	11.5	11.6	10.2	9.2	9.8

Supplementary Table S2. Klason lignin and carbohydrate monomer composition of Scots pine heartwood over 8 weeks of degradation by *R. placenta* (mg/g extractives-free wood)

	**Week 0**	**Week 2**	**Week 4**	**Week 6**	**Week 8**
**Lignin**	270.2	270.0	271.7	274.3	284.4
**Arabinose**	19.5	19.1	18.1	16.9	15.8
**Galactose**	19.3	18.4	17.0	16.6	14.9
**Glucose**	476.4	480.5	477.2	473.9	464.7
**Xylose**	71.1	71.3	67.6	68.6	64.2
**Mannose**	118.7	111.1	112.2	110.9	107.4

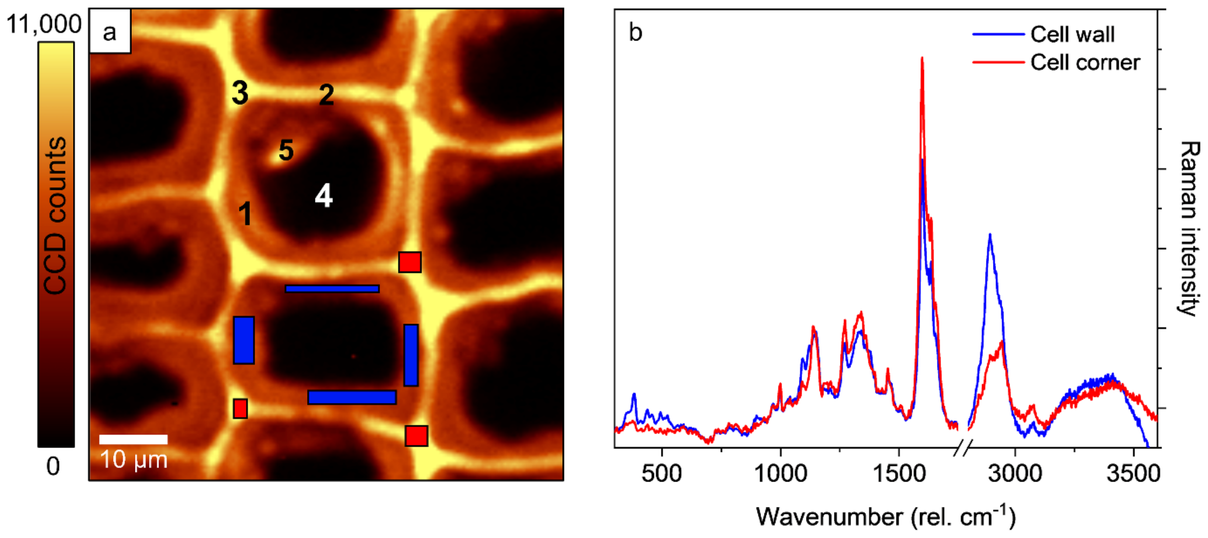

Supplementary Figure S3. Raman imaging of wood. A false-colour Raman image generated by applying a sum filter to the spectral region 1550-1700 cm^{-1} (a). The image reveals the cellular structure of pine heartwood, showing tracheid cell walls (1), middle lamellae (2), cell corners (3), cell lumens (4) and extractive deposits (5). To examine local differences in composition, average spectra can be extracted from regions of interest by selecting the relevant areas (blue and red boxes in panel a) on the image (b)

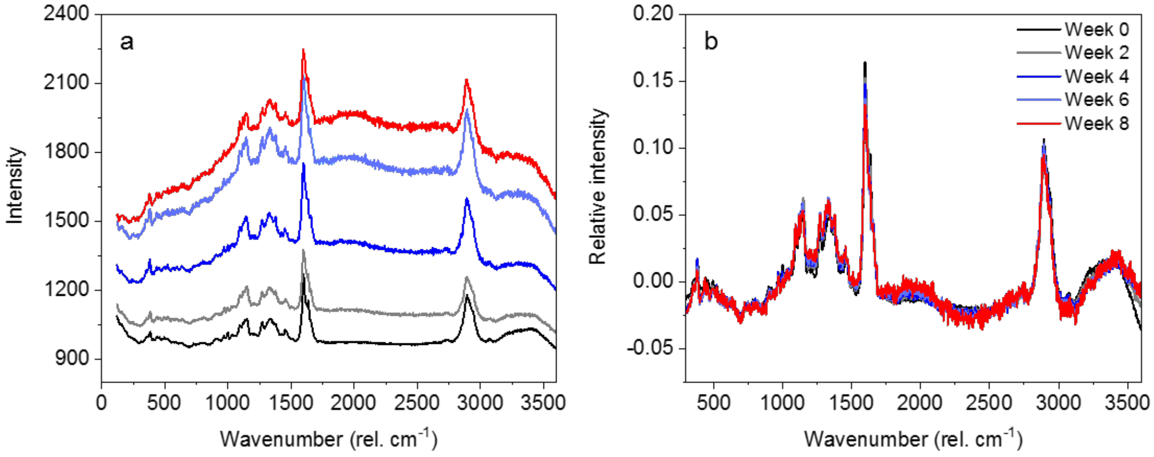

Supplementary Figure S4. Average cell wall Raman spectra before (a) and after (b) baseline correction and normalisation

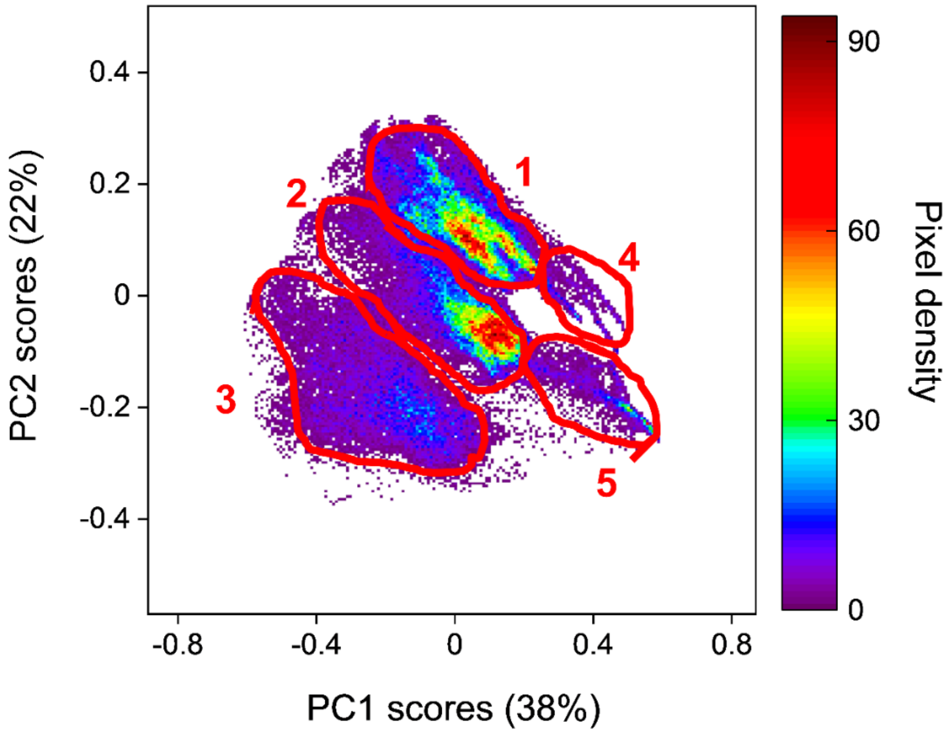

Supplementary Figure S5. PC1 vs PC2 score plot

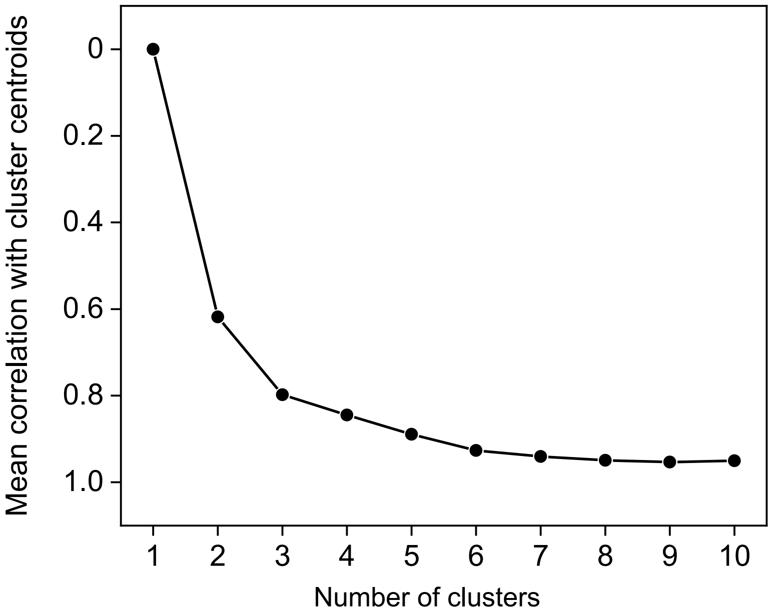

Supplementary Figure S6. Mean correlation of pixels with the respective cluster centroids at different numbers of clusters

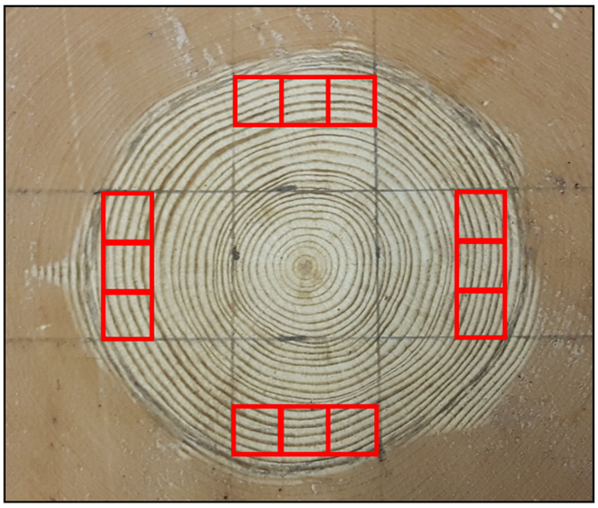

Supplementary Figure S7. Sampling of outer heartwood. Long sticks were cut from the positions indicated on the image, after which the sticks were planed and cut in length to produce the final samples
